# Conservative treatment of monolateral giant renal angiomyolipoma in a patient with Tuberous Sclerosis Complex (TSC): A case report

**DOI:** 10.1016/j.eucr.2020.101413

**Published:** 2020-09-15

**Authors:** Virgilio Michael Ambrosi Grappelli, Serena Pastore, Claudia Fede Spicchiale, Lorenzo Alteri, Andrea Turbanti, Enrico Finazzi Agrò

**Affiliations:** Urology Department, Policlinico Tor Vergata, Università Degli Studi di Roma “Tor Vergata”, Rome, Italy

**Keywords:** Giant angiomyolipomas, Tuberous sclerosis complex, Hematuria, Conservative treatment

## Abstract

Tuberous sclerosis complex has several renal manifestations like angiomyolipomas. We report a case of a giant AML and discuss its diagnosis and treatment. A 42-year-old woman was admitted to emergency department due to flank pain and hematuria. The patient had history of mental retardation and epilepsy. Abdominal CT without contrast medium revealed a large mass with a fat/blood content inside. On those findings, we diagnosed the patient a bleeding giant AML. We performed selective embolization of the bleeding source with subsequent conservative management. TSC-associated AMLs occur more frequently as multiple lesions and grow to larger size than idiopathic AML.

## Introduction

TSC has several renal manifestations including AMLs and renal neoplasms.^1^ AML occurs in 0.3% of population and comprises 3% of the solid renal masses.^2^ AMLs develop in kidneys in up to 80% of patients affected by TSC. AML associated with TSC has a higher frequency in the second and third life decades. TSC is characterized by the triad of epilepsy, mental retardation, and sebaceous adenoma. Kidney AML has a rapid growth with a significant morbidity because of the risk of rupture that leads to retroperitoneal bleeding and death. The AML management depends on tumor size.^3^ TSC clinical manifestations are different and can be life-threatening, so an appropriate surveillance and management are necessary to limit morbidity and mortality. Lots of organ systems can be involved, some during childhood and others more likely to be affected as adults.^1^ Birth incidence is estimated to be 1:58,00^2^.

## Case illustration

A 42 years old woman was admitted to our hospital due to gross hematuria and right flank pain. The patient had a history of epilepsy, delayed development, and learning difficulties during childhood. The Patient had multiple hyper-pigmented angiofibromas on her face, since her childhood. Her mother died a few years ago from chronic renal failure as a consequence of TSC.

Physical examination revealed hypotension, tachycardia, hematuria and a palpable mass at the level of the right side. The laboratory blood analyzes revealed anemia (Hb 4.9 g/dl), leukocytosis (WBC 19.100/μl), creatinine 6.07 mg/dl, azotemia 216 mg/dl, CRP 292 mg/L.

During RBC concentrates transfusion, the patient performed an abdominal CT without contrast medium that showed on the right kidney a coarse formation (17 cm × 14 cm x 9 cm) with not homogeneous density as for possible bleeding areas in the context, that entirely occupies the renal lodge and extends along the entire right flank till the right iliac fossa in the absence of safe cleavage planes with the minor structures. In addition, multiple exophytic cortical alterations were reported in the right and left kidney, some hypodense and others hyperdense, hypervascular and with adipose component in the context of dimensions ranging from a few millimeters to about 3 cm, to be referred to AMLs.

The patient was diagnosed with giant bleeding renal AML. We performed blood and clots evacuation in the bladder and bladder irrigation with three-ways Foley catheter.

**Fig. 1:**
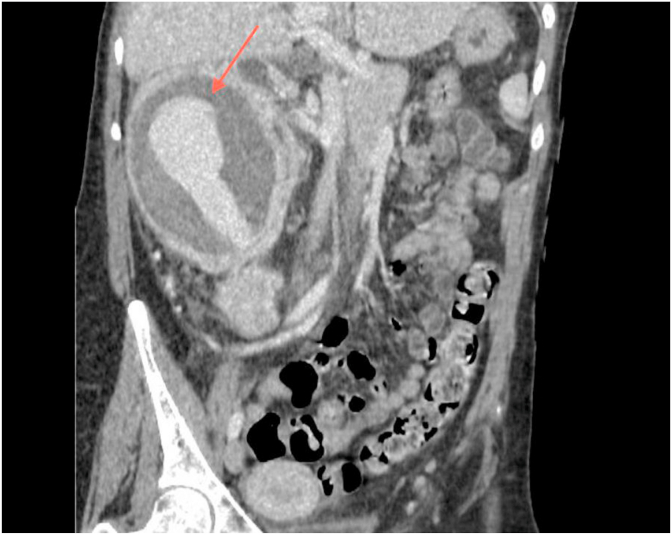
CT abdomen with contrast medium in coronal scan which in arterial phase shows active bleeding within the neoformation (red arrow). (For interpretation of the references to colour in this figure legend, the reader is referred to the Web version of this article.)

After nephrological preparation, the patient underwent a new CT with contrast medium which confirmed the information provided by the previous CT examination, highlighting a central hypervascular core borne by the known voluminous formation on the right kidney (Fig. 1).

**Fig. 2:**
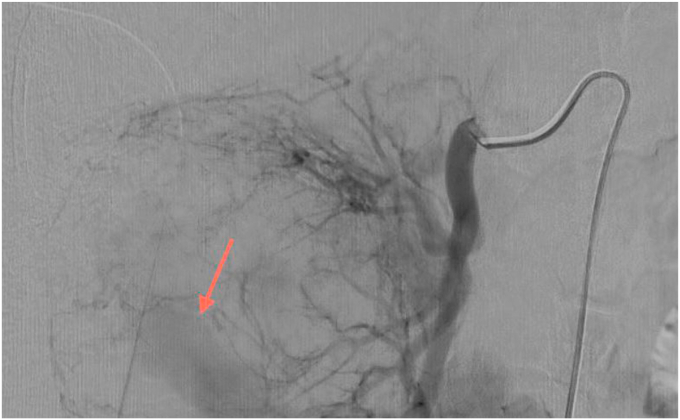
Angiography and selective embolization of the branch of the right renal artery source of bleeding (red arrow). (For interpretation of the references to colour in this figure legend, the reader is referred to the Web version of this article.)

Subsequently, the patient underwent selective catheterization of the right renal artery which showed active high-flow diffusion of contrast medium in one of the lower third division branches of the renal artery itself (Fig. 2).

Selective embolization of the hemorrhagic branch was therefore performed through metal spirals from 5 to 3 mm, distal and proximal to the blood spill, by performing a luminal trapping. The final angiographic balance showed an arrest of active blood shedding (Video 1).

Supplementary video related to this article can be found at https://doi.org/10.1016/j.eucr.2020.101413

The following is/are the supplementary data related to this article:

Video 1Angiography and selective embolization. .

The CT control performed 18 days after the procedure showed the hematoma in the right kidney substantially unchanged in size, currently less hyperdense in relation to the presence of blood component in different stages of organization.

The patient underwent a total of 6 units RBC concentrates transfusion in the first 3 days of hospitalization. No blood transfusions in the next 15 days of hospitalization, with stable hemoglobin values.

## Discussion

Most AMLs are benign and asymptomatic unless the tumor size reaches 3–4 cm or more. Lenk's triad including flank pain (53%), palpable tender mass (47%) and gross hematuria (23%) are classic symptoms in patients with tumor size more than 3–4 cm^4^. Other symptoms and signs are hemorrhage, nausea and vomiting, systemic arterial hypertension, anemia, fever, shock and urinary tract infection.

In our case, we found our patient with a tumor size more than 15 cm. Clinical manifestation were anemia due to gross hematuria, right flank pain and palpable mass in the right abdomen. The patient was diagnosed by TSC because the classical Vogt's triad symptoms were present. The presence of fat, confirmed by a negative attenuation value of −25 HU or less in CT, within a renal lesion is considered the diagnostic AML hallmark.

Management of the AMLs is related to the clinical presentation, tumor size, single or multiple lesions and potential of malignancy.^3^ Standing to the UK guidelines for managing TSC,^5^ for renal AMLs presenting with acute hemorrhage it's recommended to proceed with arterial embolization and a short course of corticosteroids as first-line therapy and making every attempt to avoid nephrectomy. For asymptomatic and growing AMLs larger than 3 cm in diameter, we should use an mTOR inhibitor as first-line therapy. For asymptomatic AMLs, selective embolization or kidney-sparing resections are possible second-line therapies. Asymptomatic patients can be treated with conservative management, with regular clinical and radiological follow-up. For those patients with pain, hemorrhage, complex lesions and enlarging tumors, nephron sparing surgery and/or intra-arterial embolization are considered to have a better outcome.

On our patient's last follow up, no hemorrhage was observed by ultrasonography in the right kidney. However, our plan is performing a nephrectomy in right kidney if significant hemorrhage occurs.

## Conclusion

AMLs associated with TSC occur more frequently as multiple lesions and reach larger sizes than idiopathic AMLs. Large and symptomatic AMLs (>4 cm) can be successfully treated conservatively/radiologically, postponing any partial/radical nephrectomy, considering the great fragility of these patients. Close laboratory tests and CT/US/MRI follow-up are needed in monitoring AMLs.

## Declaration of competing interest

The authors declare no conflict of interests.

## Abbreviations

TSC: Tuberous Sclerosis Complex
AML(s): Angiomyolipoma(s)
WBC: white blood cells
CRP: C reactive protein
RBC: red blood cells
CT: computed tomography
US: ultrasonography
mTOR: mammalian target of rapamycin
HU: Hounsfield Units
MRI: magnetic resonance imaging

## References

[1] Dixon B.P., Hulbert J.C., Bissler J.J. (2010). Tuberous sclerosis complex renal disease. Nephron Exp Nephrol.

[2] Nelson C.P., Sanda M.G. (2002). Contemporary diagnosis and management of renal angiomyolipoma. J Urol.

[3] Al-Thani H., El-Menyar A., Al-Sulaiti M. (2014). Clinical presentation, management, and outcome of patients with incidental renal angiomyolipoma in Qatar. Oman Med J.

[4] Simmons J.L., Hussain S.A., Riley P., Wallace D.M. (2003). Management of renal angiomyolipoma in patients with tuberous sclerosis complex. Oncol Rep.

[5] https://tuberous-sclerosis.org/wp-content/uploads/2019/10/SummaryofUKguidelinesformanagingTSCFINAL.pdf.

